# Differing Alterations of Two Esca Associated Fungi, *Phaeoacremonium aleophilum* and *Phaeomoniella chlamydospora* on Transcriptomic Level, to Co-Cultured *Vitis vinifera* L. calli

**DOI:** 10.1371/journal.pone.0163344

**Published:** 2016-09-22

**Authors:** Jochen Fischer, Stéphane Compant, Romain J. G. Pierron, Markus Gorfer, Alban Jacques, Eckhard Thines, Harald Berger

**Affiliations:** 1 IBWF, Institute of Biotechnology and Drug Research, Erwin-Schrödinger-Str. 56, 67663 Kaiserslautern, Germany; 2 AIT, Austrian Institute of Technology, Health & Environment Department, Bioresources Unit, Konrad-Lorenz-Straße 24, 3430 Tulln, Austria; 3 Université de Toulouse, Institut National Polytechnique de Toulouse–Ecole d’Ingénieurs de Purpan, Département des Sciences Agronomiques et Agroalimentaires, Equipe Agrophysiologie et Agromolécules, 75 voie du TOEC, BP 57611, F-31076 Toulouse Cedex 03, France; 4 Université de Toulouse, LGC UMR 5503 (CNRS/UPS/INPT), Dept BIOSYM, INP-ENSAT, 1 avenue de l’Agrobiopole, 31326 Castanet-Tolosan, France; Universidade do Minho, PORTUGAL

## Abstract

The filamentous fungi *Phaeoacremonium aleophilum* (*P*.*al*, Teleomorph: *Togninia minima*) and *Phaeomoniella chlamydospora* (*P*.*ch*) are believed to be causal agents of wood symptoms associated with the Esca associated young vine decline. The occurrence of these diseases is dramatically increasing in vineyards all over the world whereas efficient therapeutic strategies are lacking. Both fungi occupy the same ecological niche within the grapevine trunk. We found them predominantly within the xylem vessels and surrounding cell walls which raises the question whether the transcriptional response towards plant cell secreted metabolites is comparable. In order to address this question we co-inoculated grapevine callus culture cells with the respective fungi and analyzed their transcriptomes by RNA sequencing. This experimental setup appears suitable since we aimed to investigate the effects caused by the plant thereby excluding all effects caused by other microorganisms omnipresent *in planta* and nutrient depletion. Bioinformatics analysis of the sequencing data revealed that 837 homologous genes were found to have comparable expression pattern whereas none of which was found to be differentially expressed in both strains upon exposure to the plant cells. Despite the fact that both fungi induced the transcription of oxido- reductases, likely to cope with reactive oxygen species produced by plant cells, the transcriptomics response of both fungi compared to each other is rather different in other domains. Within the transcriptome of *P*.*ch* beside increased transcript levels for oxido- reductases, plant cell wall degrading enzymes and detoxifying enzymes were found. On the other hand in *P*.*al* the transcription of some oxido- reductases was increased whereas others appeared to be repressed. In this fungus the confrontation to plant cells results in higher transcript levels of heat shock and chaperon-like proteins as well as genes encoding proteins involved in primary metabolism.

## Introduction

*Phaeoacremonium aleophilum* (*P*.*al*) and *Phaeomoniella chlamydospora* (*P*.*ch*) are filamentous fungi frequently isolated from the wooden parts of grapevine (*Vitis vinifera* L.) trunks. Therefore these fungi are believed to be causal agents in disease development of the grapevine trunk disease Esca [4].

Grapevine trunk/dieback diseases are on the rise in vineyards all over the world [1][2] or [3]. However, no curative treatment for infected grapevine plants is known [5]. Outbreaks have been reported from almost all vine producing countries [6]. Typical symptoms of Esca trunk disease are discolored trunks and white rot, brown spots on fruits and “tiger stripes” on leaves [7]. In addition these symptoms can vary significantly, depending on the age of the vine, the cultivar and external factors like terroir [8][9]. Beside the two fungi, *P*.*al* and *P*.*ch* [10][11], additional species can be found in grapevine trunks: *Fomitiporia mediterranea* [12], *Botryosphaeriaceae* [13], *Eutypa lata* [14], *Phomopsis viticola* [15], *Cylindrocarpon* [16] and several others [16,17]. All these fungi belong to the phylum of Ascomycota, except the Basidiomycete *F*. *mediterranea*. Recently, the genomes of some of these species have been sequenced: *P*. *aleophilum* [18], *F*. *mediterranea* [19], *Eutypa lata* [20], *Diaporthe ampelina*, *Diplodia seriata* [21] and *P*. *chlamydospora* [22]. The actual role of these fungi in relation to trunk diseases is the topic of controversial discussions [23]. Generally, huge differences in the composition of Esca-associated fungal populations were found [4]. In addition *P*.*al*, *P*.*ch* and other fungi were isolated from affected grapevine plants showing disease symptoms (foliar ‘tiger stripes’) as well as from symptom-free host plants [24][25]. Also Bruez *et al*. [26] could not detect significant differences in fungal communities extracted from Esca symptomatic and non-symptomatic plants. These findings raised the question whether Esca is a fungal disease after all [25]. As an alternative hypotheses Hofstetter et al. discusses that Esca associated fungi may be either endophytes or saprobes. They also discuss the possibility that varieties of Esca associated fungi display different levels of pathogenicity, what cannot easily determined by using ITS sequencing or comparable methods for species identification[25]. Nevertheless internodal inoculations of *P*.*ch* and *P*.*al* in grapevine cuttings cause wood symptoms under laboratory conditions [27][28]. Therefore a Koch’s postulate regarding Esca needs to be proven. The postulate, establishing the relationship between a causative agent and the disease [29], cannot only be proved by confirming the presence of a pathogen in its host but also by measurements of its phytotoxic activity. Several studies on pathogenic behavior against plants focus on the production of extracellular cell-wall degrading enzymes and toxic metabolites [30]. For example α-glucans of different molecular weights and two naphthalene pentaketides (scytalone and isosclerone) were detected [31,32].

Plants on the other hand respond to abiotic and biotic stresses by the production of so-called reactive oxygen species (ROS) like the superoxide anion (O2•^−^), hydrogen peroxide (H_2_O_2_), hydroxyl radical (OH•) or the hydroperoxyl radical (HO2•)[33]. Pathogenic or endophytic fungi are exposed to these ROS and have developed strategies to scavenge them using either small molecules that can be oxidized (glutathione, carotenoids, flavonoids, alkaloids and ascorbic acid) or detoxifying enzymes (superoxide dismutase, peroxidase, catalase and peroxiredoxins) [34].

*Vitis vinifera* L. callus culture have been analyzed for the production of bioactive compounds [35]; hydroxycinnamic acid derivatives and anthocyanins as well as stilbene derivatives and hydroxyphenols in supernatants of the cultures were identified. Dai *et al*. [36] showed that the production of gallocatechin derivatives and flavonoids increased in grapevine callus cultures, when those were incubated with the oomycete *Plasmopara viticola*. Furthermore, the production of Quercetin-3-rhamnoside and (+)Catechin increased in calli which were co-cultured with Esca associated fungi [37].

Both fungi grow mainly in the xylem vessels and their surrounding 'cellulose-containing cell walls in grapevine’s trunk. Concerning *P*.*al*, a transformed strain *P*.*al* GFP was localized in the lumen of xylem vessel and xylem fibers six and twelve weeks post inoculation [38]. This study is consistent with the immunolocalization performed four months post inoculation [39] and also confirms microscopic observation using non-specific technics to localize fungal agents [40]. Landi *et al*. showed, that the *gfp* expression of their Pch-sGFP71 transformed line was localized in the xylem area, especially around the vessels [41]. Valtaud *et al*. showed that *P*.*ch* also invades xylem vessels[40]. Note that once established in xylem lumen and fibers both species are also able to develop in other tissues, such as the parenchyma or rays, under laboratory conditions [38] [42][40], especially in plantlets generated *in vitro* [43]. This suggests that the main nutrient supply for both fungi is provided by the xylem sap.

We attempt to decrease the complexity of interaction between *P*.*al* or *P*.*ch* and the plant in this model system by eliminating the factors nutrient depletion, water stress and the presence of other microorganisms, including bacteria that are frequently found in the grapevine trunks by using *Vitis vinifera* callus culture. This model system allows us to focus exclusively on the plant–pathogen interaction.

In order to understand how *P*.*al* and *P*.*ch* respond to the environment set by *V*. *vinifera* we analyzed the transcriptomes of both fungi in axenic or mixed cultures with *V*. *vinifera* plant cells (callus culture). We could observe that these fungi respond in a different manner to the plant cell challenge where *P*.*ch* induces detoxification and translation machinery genes and *P*.*al* alters primary metabolism and induces heat shock related genes. Nevertheless both fungi increase the transcription of oxido-reductases and we could confirm that *Vitis* leave-disks or callus culture cells react on the presence of *P*.*al* or *P*.*ch* metabolites by ROS production.

## Results

### *P*.*ch* and *P*.*al* Colonization Niches

**Fig 1:**
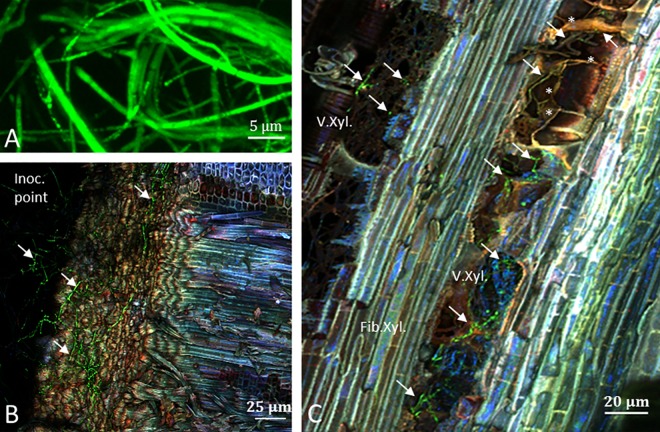
*P*.*ch*-*gfp*1 in internode 12 weeks post inoculation. A) mycelium expressing *gfp*, from a pure culture; B) Inoculation point covered with mycelium (↘) expressing *gfp*; C) Longitudinal section presenting *P*.*ch*-*gfp*1 mainly colonizing xylem vessels. (*) highlights tyloses formation in xylem vessels which partly hampered fungal spray.

First, we wanted to confirm that both fungi occupy the same niches within the grapevine trunk. Using newly transformed *P*.*ch* expressing *gfp* (Fig 1A) we analyzed the colonization niches 12 weeks post inoculation (wpi) at the internode of grapevine cuttings (cv. Cabernet Sauvignon). We could observe that the inoculation point was strongly covered with *P*.*ch*::*gfp*1 mycelium expressing *gfp* 12 wpi (Fig 1B). However, distant from the inoculation point the fungus only colonized xylem vessels and adjacent fibers (Fig 1C, S1 Fig). Also the formation of tyloses, which seemed to partly hamper fungal spray, was observed in these colonized xylem vessels. We conclude therefore that xylem vessels were the main tissue colonized by *P*.*ch*::*gfp*1 in the internode of Cabernet-Sauvignon. This confirms findings published by Landi *et al*. and Valtaud *et al*. [41][40]. Similar results were recently found by Pierron *et al*. [38] for *P*.*al*, which is also colonizing xylem vessels. Therefore, we conclude that under the same conditions both fungi occupy the same niches provided by grapevine trunks.

### A Small Proportion of Fungal Genes Are Differentially Transcribed upon Exposure to Callus Culture

We wanted to investigate the interaction of two Esca-related fungi *P*.*al* and *P*.*ch* with the plant material of *Vitis vinifera* in order to understand how these fungi adapt to this woody plant environment. Therefore, we focused on changes of the fungal transcriptome levels induced by plant defense mechanisms independently of nutrient depletion/starvation. Starvation is known to cause a wide range of cellular responses which are not environment specific and thereby hold little information about the particular plant host interaction. To avoid those interferences we designed a setup of axenic media with excess to all relevant nutrients. Thereby the fungi could grow at a relatively high growth rate and optimized conditions with or without callus culture (*Vitis vinifera* L.) added. For this reason all influences on differential gene expression should be linked to the impact of the plant/callus culture.

**Fig 2:**
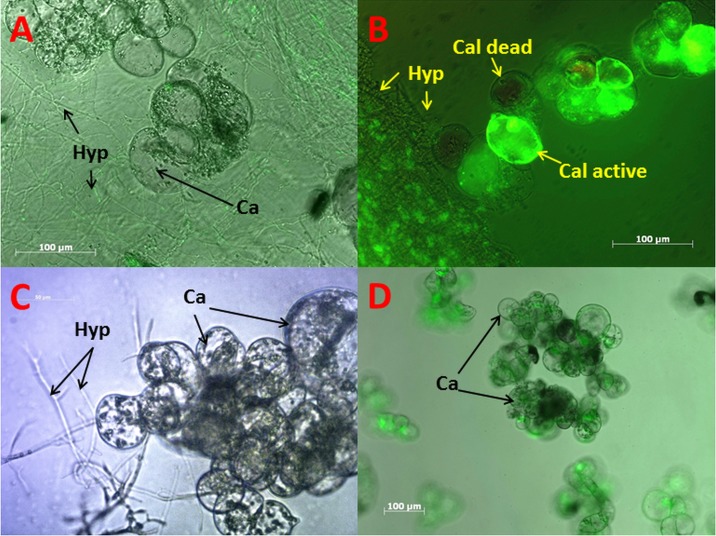
Fluorescence microscopy of *V*. *vinifera* callus culture and *P*.*ch* mycelium. A: GFP labeled *P*.*ch* visible as green hyphae (Hyp), and callus cells (Cal). B: Fluorescein-diacetate stained active callus cells and dead callus cells (none fluorescent). C: Brightfield image showing hyphae growing around callus cells. D: Monoculture of callus cells with live staining (fluorescein-diacetate).

In order to investigate the transcriptional response of the two fungi to the active plant cells we verified the viability of *Vitis* callus cultures after the incubation in co-cultures. We stained the axenic callus cultures as well as mixed cultures with fluorescein diacetate. The plant esterases of living plant cells cleave fluorescein diactetate and therefore the fluorescence would be visible under UV light [44]. We could demonstrate that up to 80% of the callus cells were viable after 36 hours of co-incubation and 60–70% of the *Vitis* cells were still metabolic active after an incubation time of 72 hours (Fig 2, S2 and S3 Figs). The GFP fluorescence of the fungal cells is a first indicator for their viability. Almost 100% of the mycelium shows the heterologous expression of the GFP fluorophore (Fig 2B) without formation of aggregates or discoloration frequently observed in dying fungal cells. Furthermore, the death of fungal cells would lead to the leakage of the gf-proteins, visible by division of proteins and the allocation of the fluorescence[45]. Unfortunately, we found that the efficiency of RNA extraction from plant cells was more than 10 times lower compared to extraction from fungal cells (although a plant RNA extraction kit, see materials and methods, was used). This was reflected by the sequencing results. Only 1 to 3% of the obtained sequences could be aligned to *V*. *vinifera* genome sequences (see Table 1). Even with a higher amount of *Vitis* cells or shorter co-incubation times the amount of isolated plant RNA could not be significantly increased so we considered only fungal sequences in this experiment. Interestingly, we could also observe in this assay that neither of the fungi is growing into the plant cells, even if the cells are already dead.

**Table 1:** Counts of sequencing reads per sample; *P*.*al* or *P*.*ch* indicate monoculture of each fungi, +VV indicates co-inoculation with *V*. *vinifera*. 1 and 2 indicate the biological repetitions.

Sample	Total Reads	Aligned to fungi		Aligned to plant	
P.al1	41599502	40089907	96.4%	147433	0.4%
P.al2	40357223	38680466	95.8%	371056	0.9%
P.al+VV1	34904228	33545735	96.1%	654957	1.9%
P.al+VV2	34619821	33088304	95.6%	1111878	3.2%
P.ch1	37154800	35081026	94.4%	129653	0.3%
P.ch2	40226826	37992439	94.4%	150335	0.4%
P.ch+VV1	34393171	31572727	91.8%	630313	1.8%
P.ch+VV2	30764387	27518852	89.5%	320653	1.0%

We sequenced the transcriptomes from both experiments, one experiment per strain of *P*. *aleophilum* (*P*.*al*) and *P*. *chlamydospora* (*P*.*ch*) with or without callus culture (*Vv*), using a HiSeq Illumina sequencer. Each sample was biologically replicated: *P*.*al*, *P*.*al+Vv*, *P*.*ch* and *P*.*ch+Vv*. Genomes from both strains are available and also gene annotations have been performed. But since this is the first transcriptome on both strains we decided to build a *de novo* transcriptome assembly based on the sequenced transcripts and the given genomes using Trinity [46].

**Fig 3:**
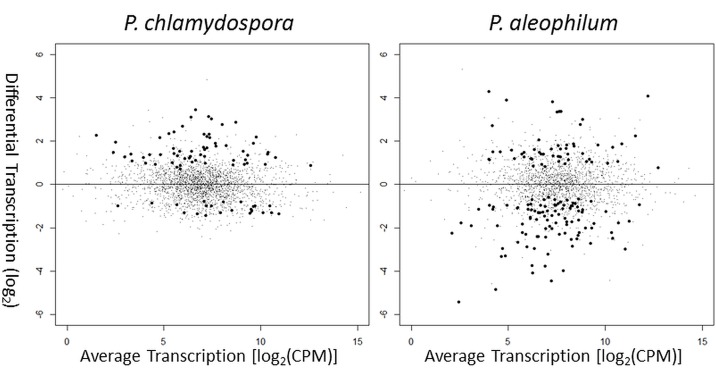
MA plot of transcriptomes. Y-axis: Differential transcription level (log2) between samples with and without addition of *V*. *vinifera* callus culture for; left: *P*. *chlamydospora*; right: *P*. *aleophilum*. Positive values indicate increased transcription in the presence of callus culture, negative values indicate decreased transcription. X-axis: average transcription levels in CPM (counts per million library reads). Black spheres indicate genes with a non-differential transcription probability (H0) of p<0.01 a.k.a. differentially transcribed; black dots indicate non-differentially regulated genes (p≥0.01).

Given a probability of non-differentially regulation (H0) we identified 85 and 153 differentially regulated genes (p<0.01) in *P*.*ch* and *P*.*al*, respectively (Fig 3). Interestingly, a low number of gene transcripts were affected by callus culture addition, especially in case of *P*.*ch*.

### Transcriptomes of Homologous Genes

**Fig 4:**
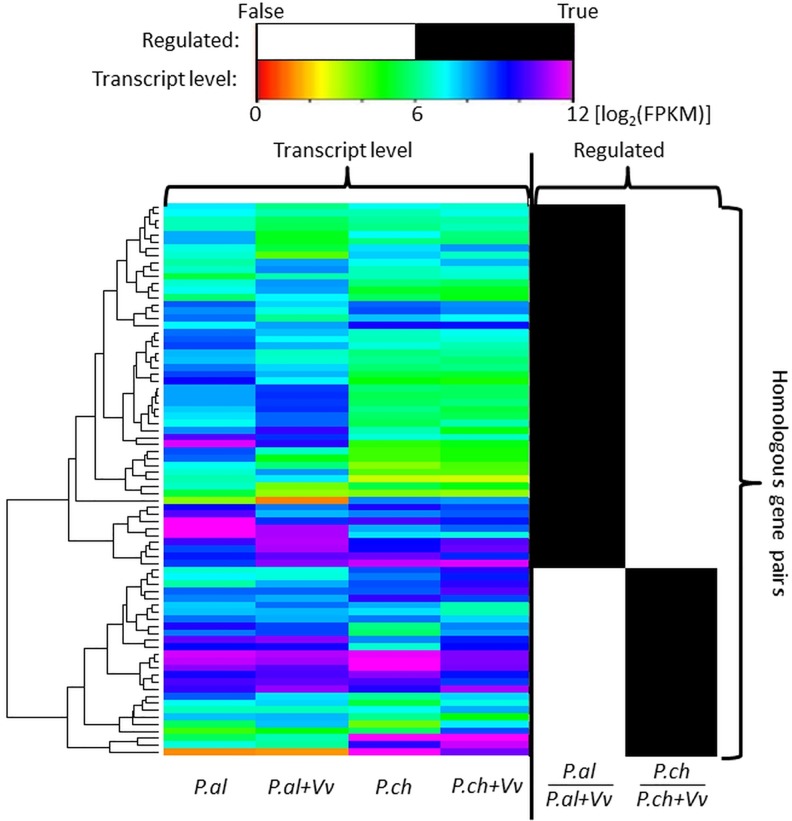
Heatmap of 79 differentially regulated homologous gene pairs (orthologous and paralogous) between *P*.*al* and *P*.*ch*; Transcript level: log2 FPKM values representing the transcription level of the 4 repeated samples *P*.*al*, *P*.*ch*, *P*.*al+Vv* and *P*.*ch+Vv*, *Vv* is indicating co-culture with *V*. *vinifera* callus. Regulated part: black indicates differential regulation (p<0.01) a.k.a. True; and white indicates non-regulated, a.k.a. False.

The two fungi *P*.*al* and *P*.*ch* are not closely related in the fungal kingdom, both belonging to the phylum Ascomycota, but *P*.*al* is of the class Sordariomycetes while *P*.*ch* is of class Eurotiomycetes. Based on the sequenced transcripts we used two sided BLASTp searches to find 837 homologous (orthologous and paralogous) gene pairs between *P*.*al* and *P*.*ch* (with e-values of less than 10E-20). In this group of genes 73 and 76 genes of *P*.*al* and *P*.*ch* are differentially regulated. These numbers are higher than expected since 7.6% (*P*.*al*) and 3.2% (*P*.*ch*) of the total transcriptome is differentially regulated with expected gene numbers in the homologous gene set of 54 and 23 for *P*.*al* and *P*.*ch*. In other words, especially for *P*.*ch* the majority of differentially regulated genes have a homologous gene in *P*.*al*. Strikingly, the set of differentially transcribed genes is strictly exclusive meaning that homologous genes that are differentially transcribed in *P*.*al* are not so in *P*.*ch* and the other way around (see Fig 4).

This is a first hint that the response mechanism due to plant cells is very different between both fungi.

Based on transcriptome data, we selected four differentially transcribed genes from each strain plus the respective beta-tubulin genes for relative quantification by qPCR to confirm our data. Results are presented in S5 Fig.

### Oxido- Reductases Are Induced in *P*. *chlamydospora* upon *V*. *vinifera* Interaction

One of the defense mechanisms of plants against microbes is the production of reactive oxygen species (ROS). It is not surprising that fungi interacting with plants need to produce enough redox equivalents to detoxify these or support the repair of ROS caused damages. One of the most highly induced genes is a putative Ferredoxin-NADP(H) reductase: PCH_01391.2 and three short-chain dehydrogenase/reductases (SDR): PCH_01570.2, PCH_01766.2., PCH_01970.2. Additionally, a NADPH-quinone reductase was identified: PCH_00455.2; a NAD-binding 2-hydroxyacid dehydrogenase: PCH_00461.2; Pyridoxamine 5-phosphate oxidase a known quencher of ROS: PCH_00427.2; a Fe-S cluster assembly protein PCH_01032.2; a Inositolphosphorylceramide-B hydroxylase for the synthesis of sphingolipids: PCH_02505.2; a Pyridine nucleotide-disulphide oxido- reductase: PCH_00915.2; a Disulfide bond forming (Dsb) protein: PCH_02049.2; a Cytochrome P450: PCH_01114.2; and a Fatty acid hydroxylase: PCH_00477.2

From these data we can clearly deduce that *P*.*ch* is reacting on *V*. *vinifera* by adaption its ability to cope with oxidative stress. The origin of this stress might be specific demands due to an altered supply for nutrients and thereby an altered metabolic flow or the production of ROS by the plant. Our experimental setup should supply the fungus with excess of nutrient so we support the second hypothesis that the production of ROS by the plant induces the observed reaction.

### Detoxifying Enzymes Are Induced in *P*. *chlamydospora* upon *V*. *vinifera* Interaction

Nitronate monooxygenase PCH_02292.2, an enzyme that oxidizes alkyl nitronates to aldehydes and nitrite and also known for detoxifying plant originated nitronates is strongly induced upon *V*. *vinifera* interaction. This is especially interesting since it was unknown that *V*. *vinifera* actually produces these compounds. Nitronates are nitroaliphatic compounds produced by a variety of leguminous plants and several microbial species and 3-nitropropionic acid is a potent inhibitor of succinate dehydrogenase, a key enzyme in the respiratory chain. It is thereby a strong toxin that may play a part in plant defense. Unfortunately, we were unable to detect these alkyl nitronates in the supernatant of *Vv*-callus or *Vv+P*.*ch* co-culture by MS (see discussion).

PCH_01677.2, another potentially detoxifying enzyme, which functions as Dienelactone hydrolase involved in biodegradation of toxic aromatic compounds, was also induced. And an Arsenical-resistance protein ACR3 which codes for a putative arsenite efflux pump: PCH_02435.2 has been induced. This is rather interesting since arsenite was historically used as treatment against trunk diseases in grapevine so one can speculate that the ability to defend against arsenite as well as its signaling remains despite application of arsenite ceased.

### Plant Cell Wall Degrading Enzymes Are Induced in *P*. *chlamydospora* during *V*. *vinifera* Interaction

Two cellulose degrading glycoside hydrolases have been identified: PCH_02658.2, PCH_02659.2; an extracellular lipase: PCH_00486.2 and a Pectinesterase: PCH_00261.2, an enzyme that degrades the primary cell-wall of plants. This indicates that *P*.*ch* is inducing plant cell wall degradation even if supply of nutrients is sufficient.

Additional genes significantly induced/repressed are listed in the S1 Table.

### Ribosomal and Translational Genes Are Repressed in *P*. *chlamydospora* by *V*. *vinifera* Interaction

In *P*.*ch*, we could detect a clear down-regulatory effect on of several genes involved in the translation of proteins. Eight ribosomal proteins were down-regulated: PCH_00610.2, PCH_01336.2, PCH_01650.2, PCH_00568.2, PCH_00335.2, PCH_01634.2, PCH_00441.2, PCH_02624.2. Additionally, an eukaryotic translation initiation factor 3 subunit D: PCH_01955.2; a eukaryotic RNA Recognition Motif containing gene: PCH_00365.2; a queuine tRNA-ribosyltransferase: PCH_01604.2; and an ubiquitin-associated domain/translation elongation factor PCH_00199.2 were repressed. These findings can be a strong hint that translation in *P*.*ch* is generally repressed upon interaction with *V*. *vinifera*.

### Oxido- Reductases Are Induced or Repressed in *P*. *aleophilum* upon *V*. *vinifera* Interaction

Similar to *P*.*ch*, in *P*.*al* some oxido- reductases are induced upon interaction with *V*. *vinifera* callus culture. PAL_01795.2, an Acyl-CoA dehydrogenase/oxidase; PAL_00101.2, an Acetyl-CoA hydrolase/transferase; PAL_00547.2, a short-chain dehydrogenase/reductase; PAL_01859.2 a Glucose-methanol-choline oxido-reductase; PAL_00629.2, a Dyp-type peroxidase; PAL_00526.2, an Epoxide hydrolase-like protein; PAL_01386.2, a Yap1 redox domain protein; PAL_00788.2, NADH:flavin oxido-reductase/NADH oxidase; and PAL_00212.2, a NAD(P)H-quinone oxido-reductase type IV are induced. Despite the fact that fewer oxido- reductase genes are induced in *P*.*al* then in *P*.*ch* there is a correlation that both fungi need to respond to the oxidative stress introduced by *V*. *vinifera* callus culture.

Interestingly some oxido-reductases are also significantly repressed under *V*. *vinifera* conditions: PAL_00450.2, PAL_01843.2 and PAL_01340.2, Short-chain dehydrogenase/reductase; PAL_01662.2, an Acyl-CoA oxidase/dehydrogenase; PAL_01151.2, an Oxoglutarate/iron-dependent dioxygenase and PAL_01001.2, a Fatty acid desaturase. Also two transcripts coding for 12-oxophytodienoate reductases are strongly repressed: PAL_01110.2 and PAL_00141.2. These proteins are part of the late steps in Jasmonic acid (JA) synthesis. It was demonstrated, that in plants Jasmonic acid (JA) is involved in regulation of plant responses to abiotic and biotic stresses as well as plant growth and development[47]. The function in fungi has to our knowledge not been a focus of major investigation.

Also several hydrolases are repressed: PAL_02006.2, PAL_01486.2, PAL_01400.2, PAL_00137.2 and PAL_00921.2, alpha/beta hydrolases; PAL_00103.2, Serine hydrolase FSH; PAL_00688.2, phosphohydrolase; PAL_00621.2 Glycoside hydrolase.

According to *P*.*ch* the *P*.*al* transcription of oxido- reductase genes is significantly altered upon *V*. *vinifera* interaction, but the pattern of the cellular response seems to be more complex. This hypothesis is supported by the altered expression of several metabolic genes described below.

### Heat Shock Proteins and Chaperones Are Differentially Transcribed in *P*. *aleophilum* during the Interaction with *V*. *vinifera* Cells

In contrast to *P*.*ch P*.*al* responds to *V*. *vinifera* with an elevating transcription of general stress response genes: a putative chaperone and retinal-binding proteins of fungi: PAL_00041.2; ubiquitinylated protein degradation Chaperonin: PAL_00562.2; a GroES (chaperonin 10)-like Alcohol dehydrogenase: PAL_00287.2. But three GroES (chaperonin 10)-like Alcohol dehydrogenase, PAL_00725.2, PAL_00151.2 and PAL_00105.2, are repressed. Also PAL_01242.2 the tailless complex polypeptide 1 (TCP-1) chaperone is repressed.

Furthermore, some genes coding for proteins with protein-protein interaction domains are strongly induced: PAL_00046.2, PAL_00047.2, PAL_00045.2, Armadillo-like; PAL_01815.2 Leucine-rich repeat and PAL_01385.2 Basic-leucine zipper.

PAL_00304.2 and PAL_00296.2 peptidases are repressed as well as PAL_01216.2 an ATP-dependent protease.

By considering the putative function in protein modification of the enzymes whose genes are differently transcribed in callus culture media we suggest that *P*.*al* responds to *V*. *vinifera* by adapting the protein maintenance machinery.

### Several Metabolic Gene Transcripts Are Differentially Regulated

Considering the very similar conditions in our experimental setup between axenic culture and callus culture we expected little impact on primary metabolism. This was in fact true for *P*.*ch* but in *P*.*al* we found several metabolic pathway gene transcripts altered.

Upregulated genes are: PAL_01632.2, Ketopantoate hydroxymethyltransferase essential for the biosynthesis of coenzyme A and penicillin production; PAL_00456.2 Dihydrodipicolinate synthetase for Lysine biosynthesis; PAL_01961.2, Prephenate dehydratase for phenylalanine and secondary metabolite biosynthesis; PAL_00051.2, N-acetylglucosamine-6-phosphate deacetylase for Galactose metabolism; PAL_01198.2, 6,7-dimethyl-8-ribityllumazine synthase in Riboflavin metabolism.

Repressed genes are: PAL_00922.2, phosphomannomutase in fructose and mannose metabolism; PAL_01940.2, maleylacetoacetate isomerase; PAL_00524.2, Inositol-pentakisphosphate 2-kinase in Inositol phosphate metabolism; PAL_00748.2, hydroxymethylglutaryl-CoA lyase in valine, leucine and isoleucine degradation; PAL_00144.2, long-chain-fatty-acid-CoA ligase and PAL_00724.2, berberine involved in the biosynthesis of numerous isoquinoline alkaloids.

In comparison to *P*.*al* in *P*.*ch* only two primary metabolic pathway genes were down-regulated (PCH_00503.2 the mandelate racemase/muconate lactonizing enzyme and PCH_00882.2 a 2,5-diketo-D-gluconate reductase) and one homoserine acetyltransferase (PCH_02447.2) was up-regulated. These results indicate, that the impact on *P*.*al* in respect to primary metabolism is much more severe.

Additional *P*.*al* genes, which are significantly induced/repressed are listed in S2 Table.

### *Vitis* Plant Cells Respond to Fungal Elicitors by ROS Production

As a validation of our results, which indicate, that the fungal cells react to the *Vitis* callus cultures ROS production, primary induced by fungal elicitors, we conducted a luminol and HRP based luminescence assay. Therefore we quantitatively measured the ROS production of callus cultures and grapevine leave disks in response to *P*.*al* and *P*.*ch* cultures and fungal elicitors of culture filtrate. Therefore we adapted the luminol-based ROS assay described by Smith and Heese[48].

The differing emission levels of activated luminol for the assorted samples were measured and compared to each other. As illustrated in S4 Fig the samples containing HRP plus luminol and water without any organisms or hydrogen peroxide showed no emission of luminol due to the oxidation by horseradish peroxidase to 3-aminophthalate via several intermediates. The positive control containing H_2_O_2_ shows the highest chemiluminenscence activity of all samples measured. The amount of oxidized luminol proportional to the amount of produced ROS by the plant cells is very similar for all samples containing fungal material and leave disk pieces as well as callus cultures, respectively. Pure fungal cultures showed no luminol activation whereas leave disk samples showed a low activation of luminol (results are illustrated in S4 Fig). This effect might occur caused by the wounding of the leave disks.

First, we had to establish the procedure to measure the ROS production by Vitis callus culture cells. Previously the assay was published for leave disks of tobacco and Arabidopsis [49,50] but the technique was not appropriate for the grapevine leaves as well as callus cultures. We tested the assay using B5VIT medium to obtain results comparable to the data of the interaction study and the transcriptome analysis. But the B5VIT medium itself caused an inhibition of luminescence except for the positive control, which was induced by H_2_O_2_. After several modifications the best result was obtained in water with the rest of medium included from the washing step. To correlate the amounts of ROS produced by the grapevine cells and the equivalent of hydrogen peroxide the positive control was diluted to a final concentration of 1 μM H_2_O_2_. By adjusting the control the counts per minute detected by the luminometer were similar to the luminescence of the samples containing fungal supernatant and plant tissue. In correlation to the samples of mRNA isolated for the transcriptome analysis the elucidation time was adjusted, additionally. Therefore we were able to show, that the expression of mRNA coding for ROS producing enzymes is with an obvious time shift correlated to the actual production of reactive oxygen species. Those ROS efflux to the surrounding medium or water, respectively. No or only a small amount of ROS production was measurable in callus cultures or leave disk pieces without contact to fungal secondary metabolites or elicitors.

## Discussion

*P*. *aleophilum* and *P*. *chlamydospora* have to date mainly been isolated from wooden parts of grapevine trunks or the trunks of fruit trees and our aim was to investigate how these two fungi adapt to this environment. We first confirmed the niches of colonization of *P*.*al* [38] and *P*.*ch* in the xylem vessels and tissues surrounding them 12 wpi. These observations corroborate with the colonization assays reported by other colleagues several months after inoculation of vine plants with *P*.*ch* and *P*.*al*. [41][39]. Since growth in wood is extremely slow and retrieval of fungal cells difficult and very inefficient we designed an experimental setup that allows good recovery of cellular material under tightly controlled conditions. We want to point out that this setup was not designed to mimic growth conditions within the trunk since despite the limited nutrient supply and overabundant cellulose/plant cell wall material conditions; additional microorganisms are present in grapevine trunks that putatively have a strong influence on the transcriptome of the respective fungi. Also the specific effect of specialized wood tissue *in planta* could not be mimicked with this setup. We wanted to clarify the influence of the plant itself on these fungi by eliminating factors involved in nutrient depletion, physical restriction of fungal growth or competing microorganisms. And we wanted to reveal whether there is an analogues response of two different fungi to the same disturbance (plant-activity).

*In vitro* callus culture setups have already been used for research on host pathogen interaction for example in Elm trees to identify Dutch elm disease resistant genotypes [51][52] or for resistant plant breeding in e.g. maize [53], potato [54] or tobacco[55]. These setups were used to identify or generate resistant plants against specific pathogens with a major focus on the plant. A similar setup to investigate the response of pathogenic fungi against eukaryotic cells has already been undertaken with *Candida albicans* invading innate immune cells [56]. Here we report a setup to investigate the response of pathogenic fungi to the plant which has not been undertaken so far to the best of our knowledge.

Since this is the first report on the transcriptome of both fungi we used *de-novo* transcriptome assembly to identify transcripts and performed functional annotation of these. This means that all functional categorizations are based on gene-homology derived predictions.

The response of *P*. *chlamydospora* seems to be more direct to the stresses applied by the plant in comparison to *P*. *aleophilum*. *P*.*ch* responses to the oxidative stress likely facilitated by the plant originating ROS by induction of oxido- reductases and reduced translation. ROS production and programmed cell death (PCD) are closely connected in plants [57] as well as in other organisms, but our fluorescein-diacetate assays showed that the majority of plant cells was still viable after 32h inoculation with either fungi indicating that the production did not take place or that the ROS produced have effectively been quenched by the fungi.

To confirm the ROS response by the plant we conducted luminol assays and measured the amount of ROS produced by the callus culture as well as grapevine leave disk samples due to fungal elicitor’s impact. Importantly, we provide necessary control experiments showing that in our artificial but efficient setup a measurable oxidative burst is accompanied by the altered transcriptomic levels of ROS disassembling enzymes. Furthermore no statistic differences were observed between the ROS production of callus cultures and grapevine leave disks after the induction of the plant cells. The luminol assay was based on the previously published method by Smith and Heese [48]. For this reason we also conducted the assay with leave disks to compare our callus culture results to the data gathered by other induced oxidative bursts of plant cells. Meanwhile, the process of ROS production by plant cells is well known and investigated [58–60]. For other fungal pathogens, in contrast to the Esca-associated fungi, it is well known that they have developed ways to sense and modify ROS accumulation in host plants. *Magnaporthe oryzae* for example has a defense suppressor 1 gene, which is a novel pathogenicity factor that regulates counter defenses against the host plant [61,62]. Another example is the biotrophic fungus *Ustilago maydis*. Its transcription factor functions as a redox sensor and controls the hydrogen peroxide detoxification systems [63]. The transcription factor is furthermore required for virulence and is responsible for preventing the accumulation of H_2_O_2_ produced by the plant NADPH oxidases. Thus, the use of transcription factors to modify the host oxidative burst could be a general strategy for fungal pathogens to cope with plant defense reactions. The detection and measurement of reactive oxygen species was thereby accomplished. Also we could show that this ROS burst did not cause PCD since the plant cells remained viable after elicitor induction. Moreover we were able to link the transcriptome data of the two fungi explored to the preceding ROS burst conducted by the fungal cells in co-culture.

More genes for plant cell wall degradation are induced in *P*.*ch* than in *P*.*al*. Interestingly *P*.*ch* induces 2 genes specific for detoxification of plant defense metabolites: Dienelactone hydrolase and Nitronate monooxygenase. Alkyl nitronates have been found in leguminous plants as a byproduct of nitrogen fixation but are not reported in *V*. *vinifera* but since no other microorganisms were present in our study we suspect the plant as their origin. The fact that we were unable to identify alkyl-nitronate in the supernatant of callus cultures (with or without fungi added) may be due to their presents in too low amounts or the repression of production under these culture conditions. Therefore, the trigger for induction of Nitronate monooxygenase would be another unknown molecule.

The relevance of *P*.*al* and *P*.*ch* for Esca disease have been discussed [23] and we wanted to shed light on one the transcriptomic aspect of putative pathogenicity. It is well established that fungi are able to produce highly bioactive substances like toxins or antibiotics, but often only under certain growth conditions [64]. We hypothesized that the disease outbreak might not only depend on the presence of the fungi but that they accessorily need to be triggered to produce toxins (e.g. secondary metabolites) that cause disease symptoms.

To our surprise we could not find any putative secondary metabolite (SM) genes regulated due to plant co-inoculation. We considered polyketide synthases non-ribosomal peptide synthases, hybrids thereof or terpene synthases as putative SM genes but could only annotate 4 in the *P*.*ch* and 2 in the *P*.*al* transcriptome, despite the fact that based on genomic gene predictions there would be more than 10 in each strain. And none of the 6 putative SM genes was differentially regulated. One explanation for this could be that a main trigger of SM induction might be nutrient depletion, a condition we did not meet in this experimental setup at any time which includes the fact that the callus culture cells differ from wood cells.

Genes putatively involved in the translational pathways were found downregulated in *P*.*ch* what could be a hint that growth in general could be reduced in the presence of plant cells. In fact in the plant colonization experiments only very slow growth was observed, 0.5-1cm in 12 weeks, compared to 1-2cm in one week on solid B5VIT medium. But naturally the main reason for slow growth in *V*. *vinifera* cuttings must be linked to limited nutrient supply in this environment.

For *P*.*al* the situation seems to be more complicated except that also *P*.*al* seems to react on oxidative stress produced by plant ROS. There is a notable impact on primary metabolic processes that cannot be observed in *P*.*ch* and in general more genes seem to be differentially transcribed in *P*.*al* (153) then in *P*.*ch* (85) despite the fact that we found more transcribed ORFs in *P*.*ch* (2661) then in *P*.*al* (2008). We want to point out that these counts of ORFs in the respective fungi are smaller than what is predicted by *ab inito* gene finding on the genomes of *P*.*al* (8926)[18] and *P*.*ch* (7279)[22], meaning that only genes transcribed in at least one condition were taken into account.

In *P*.*al* we could see that genes generally considered as coding for heat shock proteins are differentially regulated. This group of proteins is involved in translational and post translational processes and we hypothesize that this is also the main reason for the differential transcription we observe for primary metabolism genes. However, it could be the other way around that primary metabolism genes have been affected by plant metabolites and therefore a general stress response has been engaged.

Further investigation into the actual factors that drive fungal adaptation to the *in-planta* environment need to be undertaken in order to get a deeper understanding of these pathogenic fungi. The number of genes we found to be differentially regulated is surprisingly low what hints to the conclusion that the plant cells themselves don’t cause extreme stress on the fungi or trigger very aggressive measures by the fungi. These findings are coherent to the observations that *P*.*al* and *P*.*ch* can be found in healthy appearing plants [26][25] and that the outbreak of the disease may take several years. We suggest that additional factors are involved in symptom development like interaction with other fungi/bacteria or abiotic stresses like drought or nutrient depletion. From our results we conclude therefore that the two fungi, *P*.*al* and *P*.*ch*, frequently residing in the same host plant respond to the same disturbance originating from this plant respond in a very different way. We suggest that generally this is probably a perfect way for an endophytic fungal community to withstand not only plant defense mechanisms but also other biotic and abiotic stresses.

## Materials and Methods

We were allowed by Pépinière Daydé to harvest the canes in 2013 and 2014. No specific permissions were required since the material was obtained in a way that is standard procedure in vineyard work.

### *P*.*chlamydospora*-Specific Localization in Xylem Vessels

#### Fungal strains and preparation for transformation

*Phaeomoniella chlamydospora* CBS 239.74 was maintained on potato dextrose agar medium (PDA, Merck, Germany) on petri dishes placed in the dark at 26°C. Prior to transformation, a test was performed to determine whether this strain was sensitive to different concentrations of hygromycine B (25 μg.mL-1, 50 μg.mL-1, 75 μg.mL-1 and 100 μg.mL-1) in PDA medium. *P*.*ch* was transformed to obtain a strain expressing *gfp* as done in Pierron *et al*.[38] and according to Gorfer *et al*. [65].

### Plant Material

One year-old canes of *Vitis vinifera* L. cv. Cabernet Sauvignon clone 15 were harvested in January 2013 and 2014 (Toulouse, Midi-Pyrénées, France) and used to generate three eyed cuttings as described in [38]. In short, the cuttings were treated with fungicide (0.05% Cryptonol) by soaking canes for one hour and thereafter stored at 4°C until further processing. After the preparation of the canes with a bleaching bath (2.5% active chloride) for 1 min and two following rinsing-steps in water, cuttings were incubated at 4°C overnight in a solution of 0.05% Cryptonol. The water-rinsed plant material was planted in plastic trays filled with moistened autoclaved glass-wool (photoperiod 16/8, 25°C; 90% humidity). Budding and rooting took four to six weeks before cuttings were potted in 75 cL pots containing a sterile mixture of perlite, sand and turf (1:1:1 v/v) and were transferred to a growth chamber (photoperiod 16/8, 25°C; 45% humidity). The procedure was also described in Pierron *et al*.[38] (2015).

### Plant Inoculation

Cuttings (n = 20) were inoculated when at least six leaves were fully developed. Firstly, cuttings were partly surface-sterilised with a tissue sprayed with 70% ethanol. Plants were inoculated with hyphae of *P*.*ch*::*gfp*1 (n = 10) from three PDA plates. Mock-treated plants (n = 10) were inoculated with sterile PDA medium. Inoculations were performed at the internode with one syringe to ensure that the same quantity of hyphae was injected.

A wounding damage at the internode was realized (see [38]) before inoculating a cylindrical plug (3 mm long and 1 mm diameter) of *P*.*ch*::*gfp*1 or sterile PDA medium. Only hyphae in the periphery of the growing fungus were collected to avoid the danger of selecting fungal material at a different reproductive stage or with different cell activity at different locations on the same plate. After inoculation, the wound was covered with cellophane.

At sampling for microscopy, plants inoculated with *P*.*ch*::*gfp*1 or control medium (mock) were cut longitudinally or transversely with secateurs. Observations were carried out using a confocal microscope (Olympus Fluoview FV1000 with multi-line laser FV5-LAMAR-2 and HeNe(G)laser FV10-LAHEG230-2) and images were processed using ImageJ (see [66]).

For *P*.*al*, same strain and procedure as in Pierron *et al*.[38] was used.

### Fermentation, Callus Culture

The callus culture (*Vitis vinifera L*. *cv*. *Gamay Fréaux*; PC-1137), provided from the DSMZ (Leibniz Institute DSMZ-German Collection of Microorganisms and Cell Culture) grew on the recommended B5VIT medium. (http://www.dsmz.de/fileadmin/downloads/PC/medium/B5VIT.pdf). Liquid cultures grew also in B5VIT medium in 250 mL flasks filled with 50–70 mL medium. The cultures were renewed twice a week, at least after 4 days of incubation by splitting of the culture.

### Fungi Strains and Culture Conditions for Fermentation and Transcriptomics

*Phaeomoniella chlamydospora* CBS 229.95 and *Phaeoacremonium aleophilum* (*Togninia minima*) CBS 100398 were provided from the CBS-KNAW culture collection (CBS-KNAW Fungal Biodiversity Centre, The Netherlands). The strains grew on HMG medium (4 g/L yeast extract, 10 g/L malt extract, 10 g/L glucose, pH 6.5) and were transferred two new agar plates after two to four weeks. The fermentation was conducted in 50 mL HMG-liquid medium in 100 mL flasks.

### Mixed Cultures (Interaction Cultures)

Previous to the interaction studies the fungi were rose in 50 mL flasks in HMG medium inoculated with a spore suspension (1x10^5^ spores/mL) for 72 hours. Thereafter the cultures were pelletized via centrifugation (10 min, 4000 rpm, 22°C) and washed once with B5VIT medium. The aggregated mycelium was suspended in 5 mL B5VIT medium.

The callus cultures were incubated in 50 mL B5VIT medium for 6 days and then centrifuged (5 min, 1500 rpm, 22°C) and dissolved in 45 mL of fresh B5VIT medium.

The two organisms (*Vitis* callus culture and *P*.*ch* respectively *P*.*al*) were transferred to new flasks. The co-cultures were incubated for 72 hours (80 rpm, 22°C) on a rocking platform shaker.

All tests were made as triplets. Samples were the fungi-callus-culture mixtures and the fungi without callus culture in B5VIT medium. The preparation of the samples without callus culture was the same as in the co-cultures, but the pelleted mycelia were dissolved in 50 mL B5VIT medium.

### Viability Assay

The strains *P*.*al* (GFP) and *P*.*ch* (GFP) were transformed as described by Figueiredo *et al*.[67]. The strains obtained from the American Type culture collection were cultivated on minimal medium (according to Kramer *et al*. supplemented with 400mg/L Hygromycin [67,68].

The co-cultures were also monitored with a fluorescence microscope. Therefore were strains of *P*.*al* and *P*.*ch* used which contain a pCambia Vector construct that includes a Hygromycin resistance and the coding sequence for the green fluorescence protein. The green fluorescence emission associated with GFP was detected using an Imager M.2 microscope (Zeiss, Jena) with the following filter settings: 488 nm excitation and 515 nm emission. Images have been recorded with the AxioCamMRm camera.

The co-cultures were also stained with fluorescein-diacetate to determine the amount of viable grapevine callus cells. The co-cultures were monitored for 120 hours every 24 hours. To stain the preparation 20μL of cell-mixture and 2μL fluorescein-diacetate solution (1mg/10mL) were mixed. The fluorescence is only detectable if esterases in living cells cleave the fluorescein-diacetate [69].

### RNA Extraction and Sequencing

The co-incubation was stopped after 72 h. The growth medium and the mycelium or the plant cells + mycelium were separated via filtration. The mycelium/ plant cells were frozen immediately in liquid nitrogen and stored at -86°C.

RNA was extracted using Qiagen RNAeasy Plant Mini Kit and RNAse-Free DNAse Set for complete removal of genomic DNA. RNA was reverse transcribed using Biorad iScript™ cDNA Synthesis Kit and specific transcripts were verified using qPCR (iQ™ SYBR® Green Supermix, Biorad). Primers used are found in S3 Table. Total RNA samples were further processed by VetCORE, VetMed University Vienna for high throughput sequencing. RNA fragmentation and Reverse Transcription were realized according to TruSeq RNA prep Kit v2 manual (Illumina, San Diego, US) and 50bp single end sequencing was performed using HiSeq V4 Illumina sequencer. Between 30 and 40 million reads were obtained per sample.

### Data Processing

After quality control, all remaining sequences were used for genome based *de-novo* transcriptome assembly by Trinity [46]. The obtained transcripts were filtered for low coverage and mRNA, ORF and peptide sequences were calculated using the Trinity plugin Transdecoder. For *P*.*ch* and *P*.*al* 2661 and 2008 complete ORFs and Protein sequences were found as being sufficiently transcribed for detection in this experiment in at least one condition. The ORFs were mapped back on the respective genomes using BLAT [70] and gff files were constructed containing exon positions of the respective ORFs. Novoalign (Novocraft) was used for mapping the sequencing raw files onto the respective genomes and the python script HTSeq (Anders *et al*., 2014) was used to obtain coverage counts per exons. Normalization and statistics of the coverage counts per transcript were done using R/Bioconductor packages limma [71], EdgeR and voom [72] to identify differentially regulated genes using mean-variance weighting (voom) and TMM normalization (Gentleman et al., 2004). A significance cut-off of p < 0.01 (adjusted for multiple testing by the false discovery rate method) was applied for analysis. Data have been deposited at NCBI GEO under the accession number GSE67197.

The protein sequences were annotated using InterProScan [73] to receive also GO (gene ontology) and KEGG (pathway) information.

### ROS Assay

Leave disks of grapevine plants (1cm^2^) were cut into sixteen parts 24 hours before the ROS assay was conducted and placed into a 96-well plate. Each sample contained 16 pieces in a triplet. One half of the leaf sections was covered with 200 μL sterile fungal culture filtrate of Pal or Pch grown in B5Vit Medium for four days. As a control the other 50 percent of the leaf samples were covered with H_2_O. Thereafter the samples were incubated over night at 22°C. In a second approach cells of a three weeks old callus culture (which were transferred weekly in new B5Vit medium) were diluted to an optical density of OD_600_ = 0.1. Afterwards 200 μL of the dilution were centrifuged in a 1.5 mL reaction tube and the supernatant was discarded. The remaining cell pellet was resuspended in 100 μL H_2_O and transferred to a 96-well plate as well. To the callus culture cells 100 μL fungal culture filtrate was added.

The Peroxidase from horseradish (HRP, Sigma-Aldrich, #P8375) was prepared as a stock solution (500x) by dissolving 10 mg/ml lyophilized powder in H_2_O_UF_. The stock solution was stored at -20°C until usage in the test at a concentration of 20 μg/mL. Luminol was also prepared as a 500x stock solution by diluting 17 mg luminol (luminol, Carl Roth, # 4203.1) in 1 mL of 200 mM KOH. The concentration used finally in the test system was 0.2 μM. Therefore 20 μL of both stock solutions were added to 10 mL distilled water [48].

Immediately prior to the luminol assay the mixture of luminol and HRP was added to the leave samples and callus cultures (Repka, 2001). The measurement of ROS production by the *Vitis* cells as a reaction to the fungal culture filtrate was monitored without delay after the reaction solutions were added. For this purpose an EnVision Multilabel Reader (Perkin Elmer, 2104-0010A) and the standard luminescence program were used. The measurement was conducted for 12 minutes. The reaction progress was observed by measurement every 20 seconds in alteration with a 10 seconds shaking step (120 rpm). As controls fungal cells leave disks and *Vitis* callus culture cells were added separately to the reaction solutions also in the 96-well microtiter plate. Furthermore a control containing 1 μM H_2_O_2_ was conducted to determine the reactive potential of the HRP and the luminol solutions. The reaction solutions were also added as a negative control to water. All results are compiled in S4 Fig.

## Supporting Information

S1 Fig Fluorescence microscopy of P.al-GFP on grapevine trunk. P.al-GFP strain, visible as green hyphen, 12wpi in xylem vessels, indicated by arrows.
(TIF)

S2 Fig Fluorescence microscopy of *V*. *vinifera* callus culture and *P*.*al* mycelium. A: GFP labeled *P*.*al* visible as green hyphae (Hyp), and callus cells (Cal). B: Fluorescein-diacetate stained active callus cells and dead callus cells (none fluorescent). Red bar 100μm.
(TIF)

S3 Fig Viability of calli cells during co-cultivation with fungal mycelium and control in axenic culture. Light gray, co-incubation of callus cells with Pch; gray, co-incubation of callus cells with Pal and dark gray, axenic callus culture. The calculation was made based on staining the callus cells with fluorescein diactetate and count cells with a Neubauer counting camber.
(JPG)

S4 Fig Results of the ROS-Luminol Assay. Display of the transient ROS production of Vitis vinifera callus and leaf tissue cells in response *P*.*al* and *P*.*ch* culture filtrate. All values were measured using an EnVision Multibal Reader (Perkin Elmer, 2104-0010A) and a standardized luminol assay. *P*.*ch* Vitis callus is the sample where *P*.*ch* filtrate was premixed with callus culture cells. *P*.*ch* Vitis disk is the nomenclature for Vitis leaf disks that were mixed with *P*.*ch* culture filtrate preliminary to the measurement. The labeling for *P*.*al* is identically carried out. The two control reactions are the activation of the horse-reddish peroxidase by H_2_O_2_ (control hydrogen peroxide) and the buffer mixed only with luminol and HRP, which was not activated by H_2_O_2_. The emission of the activated luminol was measured every 20 seconds for 12 minutes (as can be seen at the x-axis). The measured counts per minute are arranged in analogy to all the samples conducted as triplets and in comparison to 1μM H_2_O_2_ equivalent in the control setup.
(TIF)

S5 Fig Relative transcription of indicated genes in respect to b-tubulin as determined by qPCR. Different transcription levels correlate with measurements from high throughput sequencing. +VV indicates co-cultivations.
(JPG)

S1 Table *P*.*ch* differential transcription of annotated genes. Columns: gene name, differential transcription (log2), average transcription (log2), probability to dismiss H0, gene description 1, gene description 2, GO annotation, pathway annotation.
(TXT)

S2 Table *P*.*al* differential transcription of annotated genes. Columns: gene name, differential transcription (log2), average transcription (log2), probability to dismiss H0, gene description 1, gene description 2, GO annotation, pathway annotation.
(TXT)

S3 Table Primer used for qPCR. (TXT)
