# Supplementary figures and images for: Differing Alterations of Two Esca Associated Fungi, Phaeoacremonium aleophilum and Phaeomoniella chlamydospora on Transcriptomic Level, to Co-Cultured Vitis vinifera L. calli
Source: PLoS One. 2016 Sep 22;11(9):e0163344. doi: 10.1371/journal.pone.0163344 (PMC5033417; doi:10.1371/journal.pone.0163344)

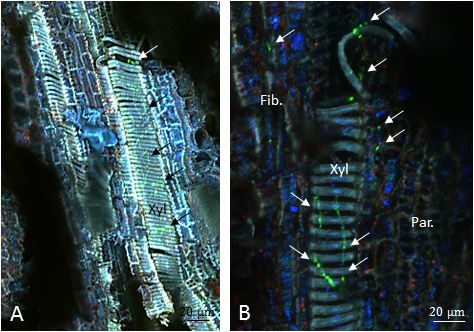

Supplement: S1 Fig — P.al-GFP strain, visible as green hyphen, 12wpi in xylem vessels, indicated by arrows. (TIF) [file pone.0163344.s001.tif]

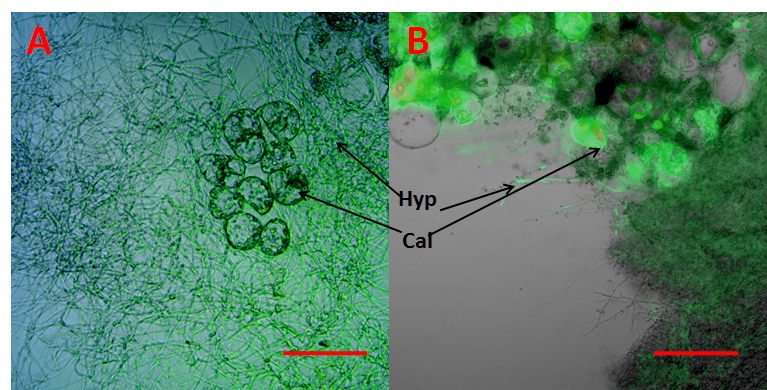

Supplement: S2 Fig — A: GFP labeled P.al visible as green hyphae (Hyp), and callus cells (Cal). B: Fluorescein-diacetate stained active callus cells and dead callus cells (none fluorescent). Red bar 100μm. (TIF) [file pone.0163344.s002.tif]

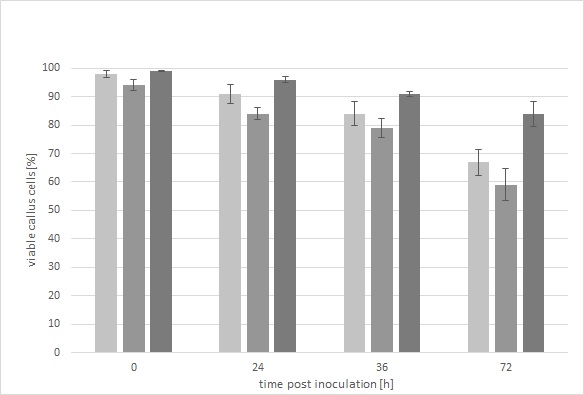

Supplement: S3 Fig — Light gray, co-incubation of callus cells with Pch; gray, co-incubation of callus cells with Pal and dark gray, axenic callus culture. The calculation was made based on staining the callus cells with fluorescein diactetate and count cells with a Neubauer counting camber. (JPG) [file pone.0163344.s003.jpg]

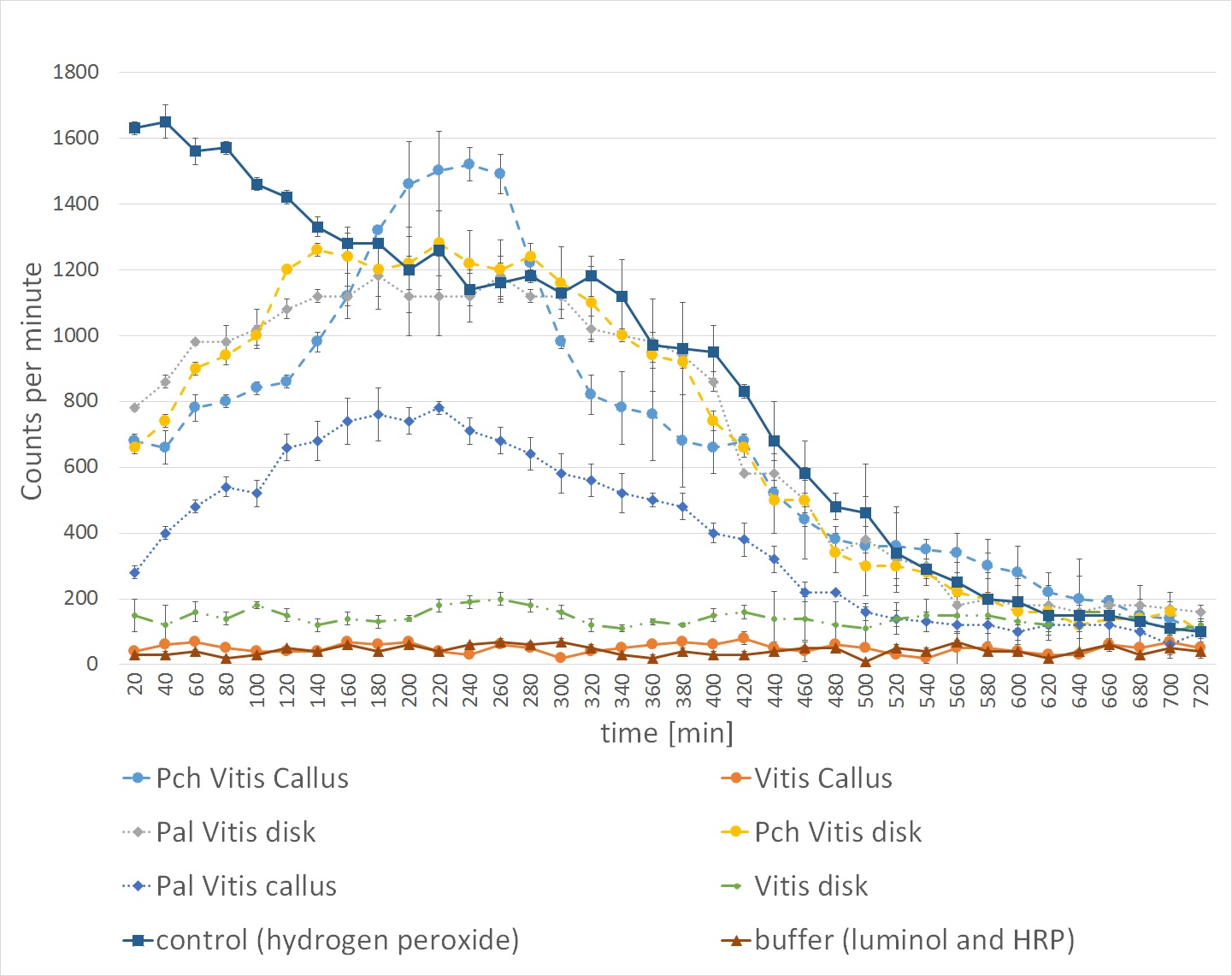

Supplement: S4 Fig — Display of the transient ROS production of Vitis vinifera callus and leaf tissue cells in response P.al and P.ch culture filtrate. All values were measured using an EnVision Multibal Reader (Perkin Elmer, 2104-0010A) and a standardized luminol assay. P.ch Vitis callus is the sample where P.ch filtrate was premixed with callus culture cells. P.ch Vitis disk is the nomenclature for Vitis leaf disks that were mixed with P.ch culture filtrate preliminary to the measurement. The labeling for P.al is identically carried out. The two control reactions are the activation of the horse-reddish peroxidase by H2O2 (control hydrogen peroxide) and the buffer mixed only with luminol and HRP, which was not activated by H2O2. The emission of the activated luminol was measured every 20 seconds for 12 minutes (as can be seen at the x-axis). The measured counts per minute are arranged in analogy to all the samples conducted as triplets and in comparison to 1μM H2O2 equivalent in the control setup. (TIF) [file pone.0163344.s004.tif]

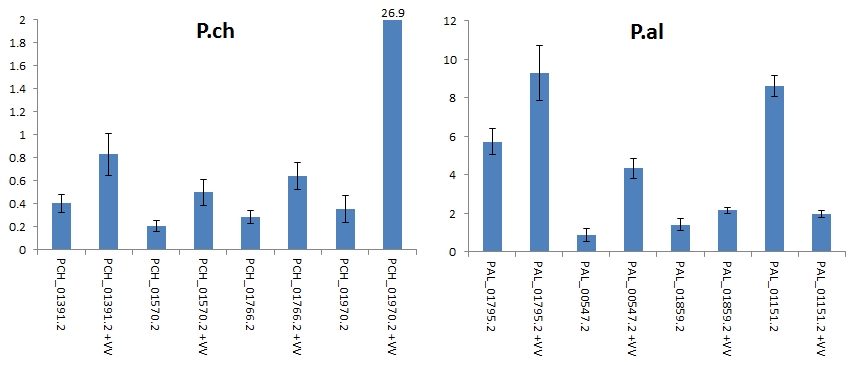

Supplement: S5 Fig — Different transcription levels correlate with measurements from high throughput sequencing. +VV indicates co-cultivations. (JPG) [file pone.0163344.s005.jpg]
